# The role of microbiota on thyroid cancer: from carcinogenicity, treatment and predictability

**DOI:** 10.3389/fimmu.2026.1912576

**Published:** 2026-10-05

**Authors:** Jian-li Han, Xin-da Lu, Jia-jia Du

**Affiliations:** 1 Shanxi Bethune Hospital, Shanxi Academy of Medical Sciences, Third Hospital of Shanxi Medical University, Tongji Shanxi Hospital, Taiyuan, China; 2 Third Hospital of Shanxi Medical University, Shanxi Bethune Hospital, Shanxi Academy of Medical Sciences, Tongji Shanxi Hospital, Taiyuan, China

**Keywords:** gut-thyroid axis, immunomodulation, microbiota, radioiodine therapy, thyroid cancer

## Abstract

In recent years, more and more attention has been paid to the mechanism of microbiota in thyroid cancer (TC). Gut microbiota (GM) and its metabolites can regulate the immune system and affect thyroid function through the gut-thyroid axis. When the GM is dysregulated, the intestinal barrier function is impaired, resulting in immune dysfunction, chronic inflammation, oncogene activation, and eventually leading to the occurrence of TC. In addition, intratumoral microbiota (ITM) is also directly involved in the occurrence, progression and invasion of TC. However, the mechanism of action between the microbiome and TC is still under exploration, and the research results in this field will have a major impact on disease cognition and clinical intervention. Chemotherapy, radiation, and immunotherapy are the main non-surgical treatments for TC. Furthermore, gut microbiota modulators, including probiotics and prebiotics, represent a potential therapeutic strategy for TC, offering new avenues for individualized treatment. In addition, the differences of microbiota in patients with TC can be used to predict tumor subtype, invasion, lymph node metastasis, and the efficacy of ^131^I treatment. This review will integrate the existing research results to explore the role of microbiota in the occurrence, development, treatment and prediction of TC, so as to provide important reference for clinical research and treatment.

## 1 Introduction

Thyroid cancer (TC) is one of the most common endocrine malignancies worldwide, of which 90% to 95% originate from thyroid follicular epithelial cells (1). According to the World Health Organization 2022 classification, TC is mainly divided into four types, among which papillary thyroid cancer (PTC) predominates, accounting for more than 85% of all cases (2). PTC generally carries a favorable prognosis, with a five-year survival rate exceeding 90% (3). Despite these favorable outcomes, the incidence of TC has been rising steadily in recent decades, prompting increasing interest in novel risk factors and mechanisms underlying its pathogenesis.

The human body harbors a vast community of microorganisms—including bacteria, fungi, protozoa, and viruses—collectively referred to as the microbiome. In a healthy state, this microbial ecosystem exists in symbiosis with the host, contributing to digestion, metabolism, and immune regulation (4). Over the past few years, accumulating evidence has revealed a complex interplay between the gut microbiota (GM) and thyroid function, giving rise to the concept of the “gut-thyroid axis”. Through this axis, the GM and its metabolites can influence thyroid hormone homeostasis, autoimmune responses, and inflammatory processes, thereby potentially contributing to thyroid carcinogenesis. Dysbiosis of the GM may result in the accumulation of metabolites that promote genetic instability in thyroid cells, ultimately facilitating tumor initiation and progression (5). Moreover, advances in sequencing technologies have enabled the identification of microbial communities residing directly within thyroid tumor tissues. This intratumoral microbiota (ITM) is now recognized as an integral component of the complex tumor microenvironment (TME) (6).

The influence of the GM on thyroid function occurs through multiple mechanisms. First, the GM plays a critical role in the absorption of certain trace elements essential for normal thyroid function (7). Second, microbial metabolites such as short-chain fatty acids (SCFAs) can regulate TC development by modulating host metabolic and signaling pathways (8). SCFAs exert pleiotropic effects, including strengthening epithelial barrier integrity and promoting immune tolerance. They contribute to intestinal homeostasis by enhancing mucus secretion from goblet cells, activating the inflammasome to promote IL-18, IL-12, and IL-17 production, stimulating secretory IgA secretion by B cells, and expanding the number and function of colonic regulatory T cells. These actions also intersect with cancer-related pathways such as AKT/PI3K/mTOR and NF-κB signaling (9). Alterations in specific microbial taxa have been observed in TC patients: for instance, Butyromonas, Escherichia coli, and Lactobacillus are altered in abundance, which can increase intestinal permeability and trigger endocrine disturbances (10). Resulting local intestinal inflammation elevates systemic levels of TNF-α, IL-1β, and nitric oxide (NO) (11). Furthermore, lipopolysaccharide (LPS) has been shown to modulate the synthesis and transformation of thyroid hormones through its effects on hepatic deiodinase activity and thyroid hormone transporter expression (12).

Standard treatment for TC includes thyroidectomy, thyroid-stimulating hormone (TSH) suppression therapy, radioiodine therapy (RAI), chemotherapy, and immunotherapy. Dysbiosis of the GM can affect the absorption and metabolism of anticancer drugs, thereby altering therapeutic efficacy. Probiotics have been shown to reduce postoperative complications and treatment-related side effects by modulating the GM (13). In addition, GM modulators such as probiotics hold promise as potential adjunctive therapies for TC.

Notably, the diversity and composition of the GM in TC patients differ from those in healthy individuals. For example, *Butyricimonas* and *Lactobacillus* are decreased in TC patients, whereas *Porphyromonas*, *Streptococcus*, and *Proteobacteria* are increased, with these differences reaching statistical significance (P < 0.05) (14). Similarly, the microbiota in TC tissues differs from that in normal thyroid tissues: *Sphingomonas* has been identified as the dominant taxon within tumors, while *Comamonas* is more abundant in peritumoral tissues (15). These distinctive microbial profiles may serve as valuable predictors of tumor characteristics and prognosis, thereby facilitating individualized clinical management.

Most studies have focused on the indirect interaction between GM and TC, and only a few studies have involved the direct carcinogenic mechanism of thyroid tissue microbiota. In addition, there is no literature that comprehensively discusses the role of microbiota in the occurrence, development, treatment and prediction of TC. By reviewing the existing studies on the relationship between microbiota and TC, this review aims to clarify the changes of microbiota and its mechanism of action in the occurrence and development of TC, which may provide new ideas for the prevention, diagnosis and treatment of TC.

## 2 Carcinogenic mechanisms of microbiota in TC

Existing studies have emphasized that microbiota has an important impact on the TME of TC mainly by regulating immune regulation and inflammatory response (16). Key microbial metabolites such as SCFAs and LPS have been identified as the core regulators of these processes, and have an impact on the synthesis and metabolism of thyroid hormones (17). As shown in **Figure 1**, these interactions suggest an important link between microbiota and TC progression. However, further studies are needed to gain insight into the underlying mechanisms.

**Figure 1:**
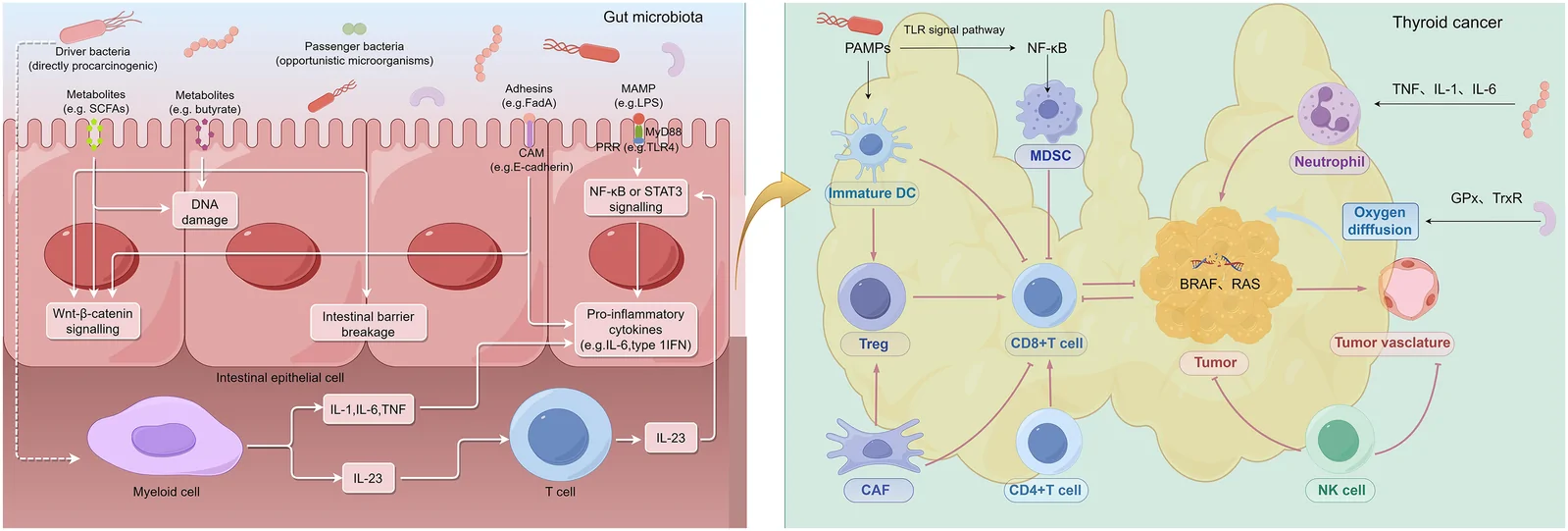
The interaction mechanism between the microbiome and thyroid cancer [Drawn by Figdraw (www.figdraw.com)]. SCFAs, Short-Chain Fatty Acids; PRR, Pattern Recognition Receptors; CAM, Calmodulin; MAMP, Microbe-Associated Molecular Pattern; LPS, Lipopolysaccharide; TLR4, Toll Like Receptor 4; NF-κB, Nuclear Factorκ-B; STAT3, Signal Transducer and Activator of Transcription; IL-6, Interleukin-6; IFN, Interferon; TNF, Tumor Necrosis Factor; IL-1, Interleukin-1; IL-23, Interleukin-23; PAMPs, Pathogen-Associated Molecular Patterns; MDSC, Myeloid-Derived Suppressor Cells; DC, Dendritic Cells; GPx, Glutathione Peroxidase; TrxR, Thioredoxin Reductase; NK, Natural Killer cell; CAF, Cancer-Associated Fibroblasts; Tregs, Regulatory T cells.

### 2.1 The diversity and distribution of GM

Most studies have shown that the diversity of GM in TC patients is decreased (18). However, some studies have shown the opposite results, Zhang et al. found that the diversity of GM in TC patients increased (14). Feng et al. through 16S rRNA gene sequencing, also found that the richness and α diversity of GM in TC patients were improved, and the *Firmicutes*/*Bacteroidetes* (F/B) ratio was significantly increased (19).

Compared with healthy controls, TC patients have significant differences in the microbial composition of the GM. SCFAs-producing bacteria such as *Butyricimonas*, *Lactobacillus*, *Faecalibacterium*, *Ruminococcaceae* and other beneficial bacteria were reduced, while harmful bacteria such as *Porphyromonas*, *Streptococcus*, *Proteobacteria* and *Neisseria* increased (14).

SCFAs are a class of small-molecule fatty acids produced by GM in the fermentation of dietary fiber, which mainly include acetic acid, propionic acid and butyric acid. SCFAs regulate energy metabolism, immune function, and inflammatory responses, and—given their established anti-tumor activity in other malignancies—are speculated to potentially inhibit TC progression, though direct evidence in TC is currently lacking.

### 2.2 Intratumoral microbiome

There are differences between thyroid tissue microbiota and GM in TC patients. The α diversity of thyroid tissue microbiota is lower, and the total amount of microorganisms in tissue samples decreases with the increase of distance from cancer tissue. Specifically, *Proteobacteria* were more common in thyroid tissue microbiota, while *Phylum Firmicutes* were more common in GM (20). The ITM was dominated by *Sphingomonas*, while the abundance of *Comamonas* was higher in peritumoral tissues (15).

ITM directly remodels the tumor microenvironment to drive TC progression via: metabolic reprogramming, metabolizing necrotic debris into energy and pro-proliferative metabolites for cancer cells; oncogenic signaling activation, where LPS activates TLR4–NF-κB/STAT3 to promote proliferation and inhibit apoptosis, and PTC-enriched genera (*Rhodococcus*, *Neisseria*, *Streptococcus*, *Halomonas*, *Devosia*) positively correlate with oncogenic drivers (BRAF, KRAS, IRAK4, CTNNB1, PIK3CA, MAP3K7, EGFR) that preferentially activate Wnt/β-catenin, PI3K-AKT, and MAPK pathways (21); immunosuppressive microenvironment induction by recruiting MDSCs and Tregs while suppressing NK and cytotoxic T cells, facilitating immune evasion; and DNA damage through bacterial genotoxins such as colibactin, causing double-strand breaks and increasing mutational risk.

Growing evidence links ITM dysbiosis to thyroid carcinogenesis, and we have systematically summarized microbial alterations in TC patients from available clinical studies (**Table 1**) and database analyses (**Table 2**).

**Table 1:** Clinical studies on the changes of microbiota and prediction model in TCs.

Year, Country	Patients	Samples	Research method	Microbiota	Prediction	Reference
2023, Portugal	37 DTCs, 10 HCs	Fecal samples before and after RAI	Shotgun metagenomics	*F. prausnitzii* ↓; After RAI: *F. prausnitzii* ↑	–	(22)
2019, China	20 TCs 18 TNs and 36 HCs	Stool	16S rRNA gene sequencing	ACE index ↑; *Neisseria* and *Streptococcus* ↑; *Butymonas* and *Lactobacillus* ↓	–	(14)
2021, China	90 TCs and 90 HCs	Stool	16S rRNA gene sequencing	Richness and diversity↓; *Proteobacteria*↑; SCFAs-producing bacteria↓	A prediction model of 10 genera distinguished TCs from HCs; A four-genus microbial signature distinguished TCs with metastatic lymphadenopathy from those without metastatic lymphadenopathy.	(18)
2019, China	30 TCs and 35 HCs	Stool	16S rRNA gene sequencing	Enrichment of 19 and depletion of 8 genera	Eight metabolites combined with 5 genera distinguished TCs from HCs	(19)
2022, China	50 TCs and 58 HCs	Stool	16S rRNA gene sequencing and an LC–MSbased metabolomics approach	Diversity and richness ↓; *Bacteroides* ↑; *g_Christensenellaceae_R-7_group*, *g_Eubacterium_coprostanoligenes_group* ↓; 27-HC, cholesterol ↓;	The combined model (three genera and eight metabolites) and the metabolite model (six metabolites) had significantly higher diagnostic validity than the microbial model (seven genera) in distinguishing TCs from HCs	(23)
2022, China	16 TCs and 10 HCs	Stool	PCR-DGGE , real-time PCR, 16S rRNA gene sequencing	Phylum level: *Firmicutes*, *Verrucomicrobia* ↑, *Bacteroidetes* ↓; Family level: *Ruminococcaceae*, *Verrucomicrobiaceae* ↑, *Bacteroidaceae*, *Prevotellaceae*, *Porphyromonadaceae*, *Alcaligenaceae* ↓; Genus level: *Escherichia-Shigella*, *Akkermansia [Eubacterium]_coprostanoligenes*, *Dorea*, *Subdoligranulum*, *Ruminococcus_2 genera* ↑, *Prevotella_9*, *Bacteroides* and *Klebsiella* ↓; Species level: *Escherichia coli* ↑	–	(24)
2023, China	60 PTCs	Fecal samples pre- and post-131I therapy	16S rRNA gene sequencing	A significant decline of F/B ratio was observed post-131I therapy.	A predictive model for response to 131I therapy with three variables including lymph node metastasis, relative abundance of *g_Bifidobacterium and g_Dorea*; *g_Dorea* was identified to be an in independent predictor of response to 131I therapy.	(25)
2021, China	19 TCs 6 TNs	Tissue samples ( tumor, para-tumor, normal tissues) and stool samples	16S rRNA gene sequencing	All sample types: *Proteobacteria* ↑; Tumor: *Proteobacteria* ↑; Stool: Phylum: *Firmicutes* ↑; Higher diversity in fecal samples than tissue; Higher diversity in tissue samples of TNs; As the distance from the TCs increased in tissue samples , the total number of bacteria dropped	–	(20)
2019, Spain	89 TCs	Tissue samples and serum samples	Serum lipoprotein profile, intratumoral cholesterol and 27-HC levels	Cholesterol and intratumoral accumulation of 27-HC promote the aggressive behavior process of PTC.	–	(26)
2021, China	30 TCs	Tumor tissues and matched peritumor tissues	16S rRNA gene sequencing	Richness and diversity ↓; Tumor tissues: *Sphingomonas* , *Aeromonas* ↑, peritumor tissues: *Comamonas*, *Acinetobacter*, *Peptostreptococcus* ↑	The combination of *Comamonas* and *Sphingomonas* could discriminate tumor samples from peritumor samples; *Sphingomonas* could distinguish between N0 and N1 stage.	(15)
2022, China	80 PTCs	Tumor samples	16S rRNA gene sequencing	The tumor bacterial diversity in patients with advanced lesions (T3/T4) was significantly higher than that in patients with relatively mild lesions (T1/T2).	A 8-taxa bacterial signature predicted PTC invasion status.	(27)
2022, China	30 DTCs with XAs, 32 DTCs without XAs following RAI after total thyroidectomy, 40 HCs	Saliva	16S rRNA gene sequencing	XAs: *porphyromonas*, *fusobacterium*, *treponema_2* ↑; Non-XAs: *streptococcus* ↓	*Fusobacterium*, and *porphyromonas* were negatively correlated with USW/SWS; *Fusobacterium*, *porphyromonas*, and *treponema_2 genera* levels were positively associated with cumulative radioiodine dose; Five genera effectively distinguished XAs from non-XAs .	(28)
2022, China	14 TCs 9 TNs and 15 HCs	Saliva	16S rRNA gene sequencing	Alpha-diversity (Chao1 and ACE) ↑; *Alloprevotella*, *Anaeroglobus*, *Acinetobacter*, *unclassified Bacteroidales*, *unclassified Cyanobacteriales* ↑	–	(29)

XAs, xerostomia; USW/SWS, unstimulated/stimulated whole salivary secretion; DTC, differentiated thyroid cancer; RAI, radioiodine therapy; F/B, Firmicutes to Bacteroides; HC, healthy control; TN, Thyroid nodules; SCFAs, Short-Chain Fatty Acids; 27HC, 27-hydroxycholesterol; PTC, Papillary Thyroid Carcinoma.

**Table 2:** Database analysis on the changes of microbiota in TCs.

Database	Sample size	Year	Method	Increase	Decrease	Reference
The GDC legacy archive	Untreated, nonirradiated tumor, and adjacent normal tissue for 563 TCs	2021	Pathoscope 2.0 software	Tumor: females: *Synechococcus sp. CC9311*; Normal Tissue : males: *Synechococcus sp. CC9311*; CPTC: *Rhodococcus fascians*; FVPTC: *Acinetobacterbaumannii AB0057*; TCPTC: *Bradyrhizobium sp. BTAi1*	Tumor: CPTC: *Rhodococcuse fascians D188* FVPTC: *Bradyrhizobium sp. BTAi1* TCPTC: *Acinetobacterbaumannii AB0057*	(30)
The MiBioGen consortium's GWAS	18340	2024	The IVW method, a reverse MR analysis	*Family Christensenellaceae*, *Family Victivallaceae*, *Genus Methanobrevibacter*, *Genus Ruminococcus2*, *Genus Subdoligranulum*, *Phylum Verrucomicrobia*	*Class Betaproteobacteria* , *Family Family XI*, *Genus Sutterella*, *Genus Ruminococcus2*	(31)
GWAS summary databases	1080 (649 DTCs and 431 controls)	2024	The MR method, a sensitivity analysis	*Class Mollicutes*, *Phylum Tenericutes*, *Genus Eggerthella*, *Order Rhodospirillales*	*Genus Eubacterium fissicatena group*, *Genus Lachnospiraceae UCG008*, *Genus Christensenellaceae R-7 group*, *Genus Escherichia Shigella*	(32)
GWAS	18340	2023	A bidirectional TSMR analysis	*Genus Butyrivibrio*, *Fusicatenibacter*, *Oscillospira*, *Ruminococcus2*, *Terrisporobacter*, *Holdemanella*	*Genus Olsenella*, *Ruminococcaceae UCG004*, *order Bacillales*	(33)
The Global Biobank Meta-analysis Initiative	6699 cases and 1620354 participants	2023	A bidirectional MR	*RuminococcaceaeUCG004 genus*, *Ruminococcus2 genus*, *Fusicatenibacter genus*, *Butyrivibrio genus*, *Olsenella genus*, *RuminococcaceaeUCG003 genus*, *Oscillospira genus*, *Streptococcaceae family*, *Holdemanella genus*	*Bacillales order*, *Proteobacteria phylum*	(10)
The Mibiogen database, GWAS summary data	211	2023	The IVW within the framework of Mendelian randomization, sensitivity analyses	*Genus Ruminiclostridium9*, *class Mollicutes*, *genus RuminococcaceaeUCG004*, *genus Paraprevotella*, *phylum Tenericutes*	*Phylum Actinobacteria*	(34)
TCGA	453 primary tumor tissue samples and 54 adjacent solid tissue normal samples	2023	The PathoScope 2.0 framework	*Botrytiscinerea*, *Pichia cephalocereana*, *Trematosphaeria pertusa*	CPTC: *Phialophora verrucosa*, *Boletinellus merulioides*, *Bipolaris sorokiniana*	(35)
GWAS	Gut microbiota data from various ethnic populations (n = 18340), TC data from an European population (n = 218,792)	2024	A bidirectional two-sample MR analysis	*Phylum Euryarchaeota*, *families Christensenellaceae*, *Victivallaceae*, *genera Methanobrevibacter*, *Ruminococcus2*, *Subdoligranulum*	*Class Betaproteobacteria*, *family FamilyXI*, *genera Anaerofilum*, *Odoribacter*, *Sutterella*, *order Burkholderiales*	(36)

TC, Thyroid Carcinoma; MR, Mendelian randomization; GDC, Genomic Data Commons; GWAS, genome-wide association studies; IVW, Inverse-variance weighted; DTC, differentiated thyroid cancer; TSMR, two-sample Mendelian randomization; TCGA, The Cancer Genome Atlas.

Several taxa showed inconsistent trends across studies. For instance, *Lactobacillus* was decreased in one cohort (14) but unchanged in another (24), while *Fusobacterium* was enriched only in the saliva of patients with xerostomia (28), not in fecal or tissue samples (15, 18). Such discrepancies may stem from technical factors, including differences in sample type, sequencing methods, primer selection, and bioinformatics pipelines, as well as from the use of relative versus absolute abundance. Biological variables such as diet, geography, disease stage, thyroid status, antibiotic exposure, and prior radioiodine therapy may further contribute. These observations underscore the need for standardized protocols and multi-center validation to distinguish robust microbial signatures from technical or population-specific variation.

### 2.3 Immune regulation

GM and its metabolites interact with intestinal lymphoid tissue and play an important role in immune regulation. When GM is dysregulated, the intestinal barrier function is impaired, leading to increased intestinal permeability and immune dysfunction (37). It can destroy the stability of the immune system, change the proliferation, apoptosis and gene expression regulation of thyroid cells, and promote the occurrence and development of TC (38).

SCFAs can up-regulate the expression of IL-22 and IL-10, inhibit the NF-κB inflammatory pathway, attenuate LPS-induced inflammatory stimulation by strengthening intestinal barrier integrity, and down-regulate the expression of TNF-α (39). Reduced SCFAs levels weaken the inhibitory effect of histone deacetylase (HDAC), and increased HDAC3 and HDAC6 levels inhibit the activation of NF-κB and peroxisome proliferator-activated receptor γ (PPAR γ), leading to congenital lymphopenia and decreased IL-22 production (40). In addition, low levels of SCFAs can hinder the promotion of intestinal regulatory T cell differentiation and the inhibition of the IL-6-STAT3-IL-17 pathway, resulting in the reduction of IL-10 secretion and the increase of IL-17 production, thereby disrupting the Th17/Treg balance (41).

Thyroid tissue microbiota may promote the release of pro-inflammatory factors through the recognition of pathogen-associated molecular patterns (PAMPs) by macrophages and the activation of TLR signaling pathway. These cytokines activate NF-κB, which in turn promotes the release of TNF, IL-1, IL-6, growth factors (GFs) and anti-apoptotic proteins, forming a microenvironment conducive to the occurrence of TC (42). ITM not only plays a key role in the progression of TC, but also may promote tumor invasion by interacting with autoimmune antibodies (27).

### 2.4 Chronic inflammation

Persistent microbiota imbalance may trigger a chronic inflammatory response (43). Chronic inflammation may promote the activation and/or mutation of multiple oncogenes (BRAF, RAS) and the inactivation of tumor suppressor genes (PTEN, PPAR γ, TP53) in thyroid tissue, thereby initiating the carcinogenic process (44).

SCFAs play a key role in maintaining intestinal mucosal health and regulating inflammation. SCFAs bind to GPR43 receptors on the surface of colon cells and inhibit the transcription of pro-inflammatory cytokines IL-1β and IL-6 by regulating the β-arrestin-2 signaling pathway upstream of the NF-κB pathway (45). In addition, SCFAs partially reduce NF-κB activity by inhibiting HDAC, thereby reducing the production of proinflammatory molecules such as IL-12, TNF-α, IL-1β, and NO (8).

In addition, LPS from gram-negative bacteria such as *Escherichia coli* and *b.fragilis* is an important trigger of chronic inflammation (46). LPS can interact with intestinal lining cells and activate immune cells to release proinflammatory mediators through TLR4/MyD88-dependent signaling pathway, thereby aggravating the inflammatory response in the intestine, increasing intestinal permeability, allowing more LPS and other inflammatory mediators to enter the systemic circulation, activating macrophages in peripheral tissues, and aggravating chronic inflammation (47). This systemic inflammatory response may increase the susceptibility of the thyroid gland to inflammation-induced injury and carcinogenesis.

### 2.5 Oxidative stress

Oxidative stress is closely related to the occurrence and development of TC. Chronic inflammation leads to the continuous production of free radicals, which may cause DNA damage and form a microenvironment conducive to tumor formation. Since the thyroid itself produces more free radicals than other glands, it is particularly vulnerable to oxidative stress, which can lead to cancer (48).

The overall intracellular activity of DUOX and NOX4 in TC cells was significantly higher than that in normal thyroid cells (49). DUOX1 overexpression will produce more H2O2, which is the main source of long-term reactive oxygen species (ROS) generation leading to persistent DNA damage and potential genomic instability (50). BRAFV600E mutation is the most common genomic alteration in TC, and the expression of NOX4 is significantly increased in the presence of BRAFV600E mutation. NOX4 can continuously produce H2O2 and hydroxyl radicals in the intracellular compartment, and also interact with the TME by recruiting tumor-associated inflammatory cells, including tumor-associated macrophages (TAMs) (51).

The REDOX homeostasis of thyroid tissue depends on antioxidant enzyme systems such as glutathione peroxidase (GPx), thioredoxin reductase (TrxR) and catalase (CAT), and *Lactobacillus* is involved in the production of these antioxidant enzymes (52). The GPx activity in most tumor tissues is higher than that in normal tissues, which may be a manifestation of the antioxidant defense system trying to counteract the excessive production of ROS. However, the expression levels of GPx1 and TrxR1 in TC cells are lower than those in normal cells, indicating that tumor tissues cannot establish an effective antioxidant system to combat the excessive generation of free radicals (53).

### 2.6 Trace elements

The imbalance of GM can affect the absorption of certain trace elements. Trace elements are involved in thyroid metabolism and carcinogenesis.

Iodine deficiency can lead to the formation of goiter or thyroid nodules, thereby increasing the risk of TC. However, studies have found that PTC is more common in regions with higher iodine intake (54), suggesting a complex association between iodine intake levels and TC. GM is involved in the process of iodine absorption and transport. GM can complete the absorption process of iodine in the stomach, duodenum and jejunum by regulating sodium/iodine symporter (NIS) (55). The reduction of butyrate-producing GM can inhibit the expression of NIS in thyroid cells, thereby reducing the iodine uptake rate, and iodine deficiency may lead to TC (56). Most T3 is produced by circulating deiodination of T4 in peripheral tissues, which is inhibited by GM (57). Additionally, iodinated contrast agents may disrupt the gut microbiota by: 1) Direct antimicrobial action: high iodine concentrations bind bacterial membrane amino acids (e.g., tyrosine, histidine) and oxidize cytoplasmic and membrane components, producing bactericidal/bacteriostatic effects that disturb microbial balance (58). 2) Altered intestinal environment: hyperosmolarity changes intestinal osmotic pressure, affecting the survival of certain bacterial populations (59).

Iron is essential for thyroid hormone synthesis, acting at both iodine uptake and utilization. The sodium-iodide symporter (NIS) mediates iodide transport into thyrocytes, and its expression is regulated by iron-dependent transcription factors; iron deficiency can downregulate NIS, impairing iodine uptake (60). Thyroid peroxidase (TPO) is a heme-iron enzyme requiring iron as a cofactor to catalyze iodide oxidation and hormone synthesis, so iron deficiency directly suppresses TPO activity (61). The gut microbiota regulates iron metabolism: *Lactobacillus* reduces Fe(III) to absorbable Fe(II), enhancing bioavailability (62). Conversely, iron availability shapes microbial composition—deficiency restricts *Enterobacteriaceae* and *Lactobacillaceae* (63), while excess reduces diversity (64). Gut dysbiosis can therefore impair iron absorption, compromising both NIS-mediated iodine uptake and TPO-dependent hormone synthesis, forming a “diet–microbiota–iron–thyroid function” axis.

Selenium levels are significantly reduced in patients with TC. Certain *Lactobacillus* species can convert inorganic selenite into selenoproteins, such as GPx and TrxR, as well as iodothyronine deiodinase, which is essential for thyroid function. Selenoproteins participate in physiological processes such as antioxidant defense, regulation of thyroid hormone metabolism, and regulation of DNA synthesis through the redox activity of the selenium atom. Low selenium levels can lead to thyroid dysfunction and even cancer (65). On the other hand, selenium can affect the composition and colonization of GM (66), and decreased serum selenium concentration is associated with higher stages of TC (67).

### 2.7 Vitamins

Vitamin C modulates the gut microbiota toward a favorable profile—increasing α-diversity and the Firmicutes/Bacteroidetes ratio and promoting SCFA production—which may support antitumor immunity relevant to thyroid cancer. Directly in thyroid tissue, vitamin C induces ROS-dependent cytotoxicity in PTC cells, inhibits MAPK/ERK and PI3K/AKT signaling in both BRAF-wildtype and mutant TC cells, and triggers ferroptosis in anaplastic TC cells (68).

Vitamin D reshapes the gut microbiota by promoting beneficial bacteria such as Bacteroides fragilis, thereby positively modulating anti-thyroid cancer immunity (69). Moreover, its anti-inflammatory, antioxidant, and DNA damage repair properties directly contribute to the prevention of thyroid cancer (70).

Vitamin A maintains a healthy gut microbiota by preserving intestinal barrier integrity and defending against pathogens, which may attenuate inflammation-driven promotion of thyroid cancer (71). Importantly, vitamin A enhances iodine uptake and NIS activity in TC cells, and retinol derivatives have been used to treat radioiodine-refractory differentiated thyroid cancer (RAIR-DTC) (72).

### 2.8 Thyroid hormone synthesis and metabolism

The LPS produced during the metabolic process of GM, especially gram-negative bacteria, play a significant role in the synthesis and metabolism of thyroid hormones. LPS can enhance TSH-induced iodine uptake and increase the expression of NIS protein, thereby promoting thyroid hormone synthesis (73). In addition, enzymes produced by GM, such as sulfatase and β-glucuronidase, play a crucial role in the peripheral metabolism of thyroid hormone (74). LPS can inhibit intestinal sulfatase activity, thereby impairing the reactivation of thyroid hormone from its inactive sulfated conjugates and reducing circulating levels of active T3 and T4 (12). This sustained disruption of thyroid hormone homeostasis can lead to a systemic hypothyroid-like metabolic state characterized by reduced basal metabolic rate, lipid accumulation, and oxidative stress imbalance. These metabolic disturbances, in turn, compromise DNA damage repair and cell cycle regulation while promoting chronic pro-inflammatory signaling, thereby creating a permissive environment for thyroid tumorigenesis (75).

Feng et al. found that flavonoids were negatively correlated with Megamonas abundance in TC patients. Flavonoids can interfere with thyroid hormone synthesis by acting as alternative substrates for thyroid peroxidase (TPO); during their enzymatic oxidation, flavonoids generate reactive quinones or other electrophilic intermediates that covalently bind to TPO’s heme prosthetic group or surrounding amino acid residues, leading to irreversible structural modification and inactivation of the enzyme. Additionally, flavonoids may directly compete with iodide and tyrosine residues for the active site of TPO, further inhibiting its catalytic activity. This combined inhibition reduces thyroid hormone production, induces a compensatory increase in serum TSH levels, and thereby promotes the development of TC (19).

### 2.9 Microbial metabolic pathways

GM may affect the TC microenvironment through metabolic pathways such as pyruvate, fatty acid metabolism, glycolysis or gluconeogenesis, thereby promoting the occurrence of TC (10).

*g_Christensenellaceae_R-7_group* and *g_Eubacterium_coprostanoligenes_group*, which are essential for lipid metabolism homeostasis, were significantly reduced in TC. While *Bacteroides*, which is associated with high-fat diet and promotes the occurrence of TC, was relatively increased (23). Previous studies have shown that cholesterol and 27-Hydroxycholesterol (27-HC) enhance the aggressiveness of TC (26). In recent years, Lu et al. found in the microbial-metabolite network that 27-HC, a lipid metabolite of *g_Christensenellaceae_R-7_group*, may promote TC proliferation through estrogen receptor drive (23). GM has a certain regulatory effect on ANGPTL4, ANGPTL4 is a fasting induced adipokine in the intestinal epithelium and plays a positive role in promoting the development of PTC (76).

The GM is also involved in the metabolism of amines and secondary bile acids. It has been reported that histamine, an amine metabolite, can promote TC formation by inducing tumor cell proliferation and tumor growth (19).

GM diversity is reduced in postmenopausal women, indicating that the gut microbiota is closely involved in estrogen metabolism (77). Mechanistically, the GM produces β-glucuronidase, which deconjugates estrogen glucuronides in the gut, releasing active estrogens back into the enterohepatic circulation and thereby elevating circulating estradiol levels. Given that estrogen acts as a potent growth factor for PTC cells by activating estrogen receptor–mediated proliferation and anti-apoptotic pathways (78), GM dysbiosis may fuel PTC progression through this estrogen-related metabolic axis.

### 2.10 Diet

Diet profoundly influences thyroid cancer (TC) risk by shaping the gut microbiota and thereby modulating metabolism, immunity, and inflammation in a tumor-relevant manner (79) (**Figure 2**). For example, a Western dietary pattern, characterized by high animal protein and saturated fat intake, has been associated with reduced Bifidobacterium abundance, and the observation that Prevotella levels are low in TC patients suggests a link between animal-derived food consumption and TC (80, 81).

**Figure 2:**
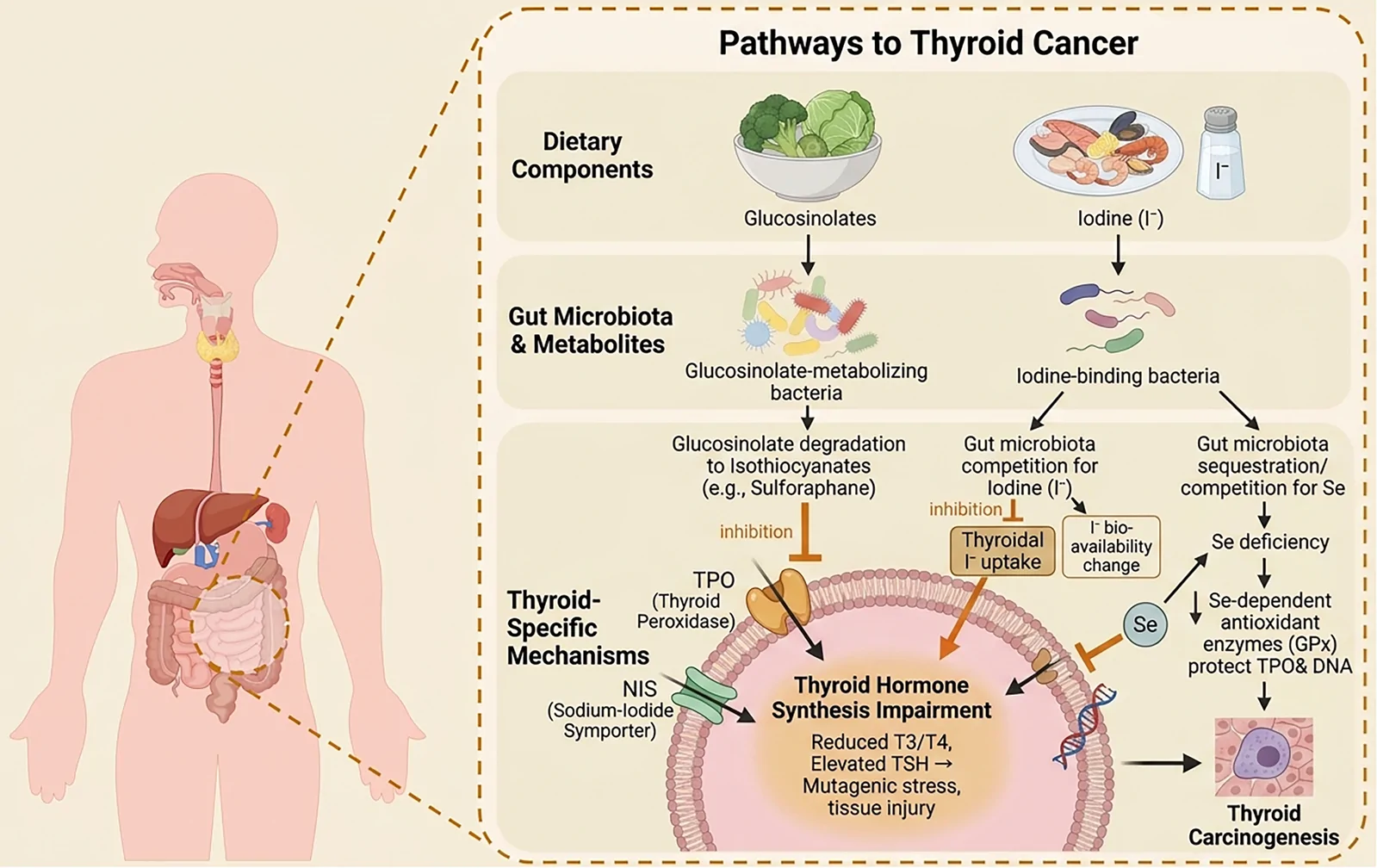
The carcinogenic effect of diet on thyroid cancer [Drawn by Figdraw (www.figdraw.com)].

The Mediterranean diet, rich in fruits, vegetables, whole grains, and olive oil, favors a gut microbiota profile that may lower TC risk (82). Olive oil phenolic compounds directly protect thyroid function through antioxidant activity and block tumor cell proliferation by inhibiting ROS generation and enzymes such as COX-2 and 5-LOX (83). Moreover, the high fiber content of this diet helps maintain gut microbial balance and promotes immune regulation that reduces TC risk (84).

Dietary polyphenols, which are compounds abundant in tea, coffee, fruits, vegetables, and whole grains, also contribute to TC protection. The gut microbiota plays an essential role in polyphenol metabolism, while polyphenols in turn restore microbial richness and improve dysbiosis, increasing beneficial bacteria such as Lactobacillus and Akkermansia (85). Importantly, a higher intake of polyphenols and phenolic acids is inversely associated with differentiated thyroid cancer (DTC) risk, especially in overweight or obese individuals (86). In addition, a case–control study found that high blood concentrations of caffeic acid, 3, 4-dihydroxyphenylpropionic acid, and ferulic acid were associated with a reduced risk of papillary thyroid cancer (PTC) in women (87).

### 2.11 Obesity and insulin resistance

Unhealthy diets, especially those high in processed foods and saturated fats, are associated with obesity (88), and the endocrine and inflammatory changes resulting from obesity are recognized risk factors for TC (89).

Obese individuals on a high-fat diet often have elevated levels of gram-negative bacteria, which produce LPS that trigger chronic intestinal inflammation (90). This increased inflammation may disrupt the integrity of the intestinal barrier, allowing bacteria to enter the bloodstream, leading to chronic inflammation of adipose tissue. Chronic inflammation often triggers insulin resistance, which is associated with increased TC risk (91).

Insulin resistance, in turn, leads to increased levels of insulin-like growth factor-1 (IGF-1), which is overexpressed in TC. High concentration of IGF-1 can accelerate the development of cancer by promoting cell malignant transformation and inhibiting apoptosis. The signaling pathway activated by IGF-1 will further increase the risk of TC (92). In addition, interference of IGF axis by high insulin levels may promote TC progression (93). Previous studies have found high expression of IGF-1 and its receptor in TC patients, indicating that IGF-1 promotes tumor proliferation by stimulating TSH to activate AKT and Raf-1/MEK/ERK signaling pathways (94).

The carcinogenic mechanisms discussed above offer a compelling framework linking the microbiota to TC. However, most functional evidence for SCFAs, LPS, and microbial estrogen metabolism derives from non-TC tumors; their role in TC remains largely hypothetical and awaits direct experimental validation in TC-specific models. Similarly, the causal relationships behind observed microbial dysbiosis in TC, including molecular mediators such as ANGPTL4/AKT and bile acid signaling, have not been confirmed through interventional studies. These proposed pathways should therefore be regarded as working hypotheses rather than established facts. Future work must prioritize longitudinal prospective cohorts, gnotobiotic TC animal models, and functional multi-omics integration to move beyond correlation and translate the gut–thyroid axis into clinically actionable strategies.

## 3 Influence of GM on the treatment of TC

Chemotherapy, radiation, and immunotherapy are the main non-surgical treatments for TC. The gut microbiota can influence TC treatment outcomes through multiple mechanisms, including bacterial translocation to tumor sites, modulation of systemic and local immune responses, metabolic transformation of therapeutic agents, enzymatic degradation of drugs, and treatment-induced alterations in microbial diversity and community composition (95). Regulating the balance of GM can improve the sensitivity of patients to TC treatment and reduce adverse reactions during treatment (13). The differences in the composition of GM in patients can explain the differences in treatment response, providing a way for individualized medicine (96).

### 3.1 Chemotherapy

Chemotherapy, which is reserved for a minority of advanced, radioiodine-refractory TC patients. The regimen of paclitaxel combined with carboplatin has been applied in the clinical treatment of advanced or refractory thyroid cancer (TC), yet this combination can directly damage the intestinal mucosa and disrupt the gut microbiota (GM) composition (97). Consequently, targeted modulation of the GM represents a promising strategy to enhance the therapeutic efficacy and mitigate the gastrointestinal toxicity of this chemotherapeutic regimen in TC patients. In TC specifically, GM dysbiosis has been linked to resistance to paclitaxel and carboplatin, and supplementation with certain bacterial species may restore tumor sensitivity to these agents (98).

### 3.2 Radioiodine therapy

RAI is a common method for the prevention and treatment of postoperative recurrence of TC, and its efficacy depends on the ability of cancer cells to take up iodine. GM affects the prognosis of radioiodine-refractory (RAIR) PTC by regulating the expression of NIS, thyroglobulin (Tg) and TSH levels (99). Notably, *in vitro* experiments have confirmed that SCFAs, such as butyric acid, can inhibit HDAC and reactivate NIS expression in TC cells, thereby promoting iodine uptake and cell redifferentiation and improving RAI efficacy (56).

Ionizing radiation during RAI can destroy the intestinal barrier, leading to bacterial translocation and triggering inflammation and immune responses (100). Studies have shown that PTC patients treated with ^131^I have a significantly reduced abundance of GM compared with healthy individuals (101), with significant decreases in *Firmicutes*, (including *Faecalibacterium*, *Lachnospira* and *Lachnospiraceae NK4A136 group*), while *F. prausnitzii* and *Bacteroides* (such as *Prevotellaceae*, *Veillonellaceae* and *Prevotella_9*) were relatively increased (25). Further studies conducted by Lapiere et al. showed that *F. prausnitzii* could protect the intestinal barrier from radiation exposure by regulating intestinal permeability, enhancing epithelial cell renewal, and preserving differentiated epithelial cells, thereby maintaining its function. In addition, *F. prausnitzii* has anti-inflammatory and immunomodulatory properties by producing SCFAs, which contributes to the regulation of the intestinal microenvironment by regulating cell signaling pathways and adjusting redox balance. This modulation may reduce the inflammatory response induced by RAI and increase sensitivity to the treatment (22).

### 3.3 Immunotherapy

Immunotherapy has emerged as a novel therapeutic option for advanced thyroid cancer (TC), particularly for patients refractory to conventional radiotherapy and chemotherapy. The gut microbiota (GM) can modulate both the efficacy and toxicity of immunotherapy in TC patients. In TC, alterations in GM composition have been linked to resistance to immune checkpoint inhibitors (ICIs), and supplementation with specific bacterial strains may restore tumor sensitivity to these agents (98). A recent case report directly demonstrated that in a patient with undifferentiated TC treated with the PD-1 inhibitor pembrolizumab, the abundances of Bacteroidales (especially Bacteroidaceae and Rikenellaceae) and Clostridiales (such as Ruminococcaceae, Veillonellaceae, and Lachnospiraceae) increased markedly, along with a significant rise in α diversity, suggesting that the microbiome actively participates in modulating the immunotherapeutic response in TC (102). Bacterial taxa that are positively correlated with anti-tumor immune factors, including Bacteroidetes, Akkermansia, and Lactobacillus (103), may therefore represent candidates for enhancing anti-tumor immunity in TC patients receiving immunotherapy.

Moreover, the incorporation of the CTLA-4 antibody gene into Salmonella typhimurium has shown potential to improve drug delivery efficiency and therapeutic outcomes (104), offering an innovative delivery strategy for TC immunotherapy.

Importantly, regarding toxicity, the GM can mitigate immune-related adverse events; specifically, the presence of Bacteroidetes or Bifidobacterium has been shown to reduce the incidence of colitis following ipilimumab treatment (105), a protective effect that could be leveraged to improve the safety profile of immunotherapy in TC patients.

## 4 Therapeutic measures related to GM

GM regulators such as probiotics and prebiotics can restore GM imbalance, protect intestinal barrier function, reduce inflammation level, and restore immune system homeostasis. It has become a potential individualized treatment method for TC, and has a positive effect on regulating thyroid homeostasis and systemic metabolic state. We summarized the existing clinical studies on the effects of probiotics, vitamins and trace elements on TC patients (**Table 3**).

**Table 3:** Clinical study on the effects of probiotics, vitamins and trace elements on TCs.

Year, Country	Study	Patinets	Supplements	Results	Reference
2022, China	Prospective analysis	50 TCs undergoing thyroidectomy	Probiotics (*Bifidobacterium infantis*, *Lactobacillus acidophilus*, *Enterococcus faecalis*, *Bacillus cereus*)	Probiotics supplementation can significantly reduce the incidence of THW symptoms and lipid levels, and improve intestinal and oral microbial diversity in postoperative patients with thyroid cancer undergoing THW before RIT	(13)
2022, Brazil		46PTCs	Vitamin A and vitamin E	Lower ROH serum levels were more likely to present lymph node metastasis and/or angiolymphatic invasion; Higher vitamin A and vitamin E intake are related to the absence of extrathyroidal extension and lymph node metastasis, respectively.	(106)
2019, Germany	Prospective analysis	13 RAI-R PTCs were treated with 13-cis-RA followed by iodine-131 treatment	Vitamin A	RAI-R PTCs with BRAF V600E receiving redifferentiation with RA and RIT showed response.	(107)
2023, Republic of Korea		9,739 DTCs who underwent thyroidectomy	Vitamin D	Vitamin D intake reduced the risk of all-cause and total cancer mortality but had no effect on thyroid cancer mortality.	(108)
2022, China	Prospective analysis	460 DTCs who treated with 131I	Vitamin C	Vitamin C supplementation has a protective effect on salivary glands in DTCs after 131I treatment.	(109)
2023, China		187 LLNM PTCs and 289 NLLNM PTCs	Iodine	The nomogram model based on iodine nutrition and other clinicopathological features is effective for predicting the lateral lymph node metastasis in PTCs.	(110)
2017, Turkey	Prospective analysis	30 TCs who underwent operation and 20 HCs	Zinc and selenium	Changes in the serum and thyroid tissue levels of trace elements like zinc and selenium, which play a critical role in thyroid function, might be associated with the pathogenesis of thyroid cancer.	(111)
2017, Korea		16 DTCs were treated with total thyroidectomy followed by 131I treatment	Selenium	Selenium supplementation during 131I treatment was effective to reduce salivary glands damage by 131I radiation in DTCs.	(112)

TC, Thyroid Carcinoma; PTC, Papillary Thyroid Carcinoma; ROH, retinol; RAI-R, radioiodine-refractory; RA, retinoic acid; RIT, radioiodine therapy; DTC, differentiated thyroid cancer; LLNM, lateral lymph node metastasis; NLLNM, non-lateral lymph node metastasis; THW, thyroid hormone withdrawal; HC, healthy control.

### 4.1 Probiotics

Probiotics are defined as live microorganisms that, when administered in adequate amounts, confer a health benefit on the host. Their anti-tumor mechanisms in the context of TC are multifactorial: 1) they can directly supplement beneficial bacteria capable of producing short-chain fatty acids (SCFAs), such as Lactobacillus and Bifidobacterium species, which promote cancer cell apoptosis and reduce oxidative stress (61); 2) they competitively exclude pathogenic bacteria and enhance intestinal barrier function by upregulating the expression of tight junction proteins, thereby reducing the translocation of pro-inflammatory microbial products like LPS and lowering serum endotoxin levels (113); and 3) they regulate both systemic and local immune responses, mitigating treatment-related inflammation and improving intestinal health, which can assist chemotherapy and immunotherapy (61). Specifically, supplementation with a probiotic mixture containing Bifidobacterium has been shown to reduce chemotherapy-induced mucositis and radiation-induced diarrhea (105). In the specific setting of post-thyroidectomy patients experiencing thyroid hormone withdrawal (THW) before radioiodine therapy, a regimen containing Bifidobacterium infantis, Lactobacillus acidophilus, Enterococcus faecalis, and Lactobacillus infantis significantly decreased the incidence of THW symptoms (fatigue, constipation, weight gain, and dry mouth), lowered serum lipid levels, and increased the diversity of both intestinal and oral microbiota (13). Furthermore, these probiotics can modulate plasma lipid metabolism through SCFA-mediated signaling pathways (113). Despite their benefits, excessive use of certain probiotics may pose risks in susceptible individuals, such as Lactobacillus-associated bacteremia and liver abscess formation (114), highlighting the need for careful strain selection and patient risk stratification.

### 4.2 Prebiotics

Prebiotics are non-digestible food ingredients that selectively stimulate the growth and/or activity of beneficial gut bacteria, thereby conferring a health benefit. Common prebiotics include inulin, oligosaccharides, oat β-glucans, and galacto-oligosaccharides (115). Mechanistically, prebiotics serve as substrates that are fermented by commensal bacteria—particularly butyrate-producing species—to generate SCFAs, which in turn mediate anti-inflammatory and immunomodulatory effects along the gut–thyroid axis. For instance, inulin has been demonstrated to significantly increase the abundance of beneficial bacteria such as Bifidobacterium and Akkermansia, leading to enhanced SCFA production and consequently improved thyroid function and reduced TC risk (116). A major advantage of prebiotics over probiotics is their non-living nature, which eliminates the risk of infection in immunocompromised patients and allows them to exert biological effects without requiring live bacterial colonization of the host gut (117).

### 4.3 Postbiotics

Postbiotics encompass inactivated microbial cells and/or their bioactive metabolites that confer a health benefit when administered in adequate amounts. They offer favorable safety and stability profiles compared to live probiotics. Among the most well-studied postbiotics are SCFAs, which are produced through bacterial fermentation of prebiotics and can directly exert anti-inflammatory and immune-modulating functions (117). Butyrate, a representative SCFA, not only alleviates the side effects of chemotherapy and reduces oxidative stress but also improves thyroid function. Mechanistically, SCFAs act as signaling molecules by activating G protein-coupled receptors (GPRs, such as GPR41 and GPR43) and inhibiting histone deacetylases (HDACs), thereby modulating gene expression to promote an anti-inflammatory and anti-tumor microenvironment (118). In the context of TC, postbiotics show considerable potential as adjuvant therapeutic options: they help maintain intestinal barrier integrity, beneficially modulate the gut microbiota composition, enhance the host’s anti-cancer immune response, and suppress chronic inflammation, all with a minimal profile of adverse reactions (119).

### 4.4 FMT

Fecal microbiota transplantation (FMT) is a novel method for regulating GM, which has shown great promise in clinical trials. In malignant melanoma, transplantation of fecal microbiota from PD-1 inhibitor responsive patients to drug-resistant patients can significantly improve the prognosis (120), suggesting that FMT can also be used for TC treatment, and more clinical studies are needed to provide evidence for reference.

## 5 The predictive value of the microbiota in TC

Several studies have characterized the microbiota in feces or tissues of different subgroups of TC patients with the use of genetic sequencing technology to predict tumor subtype, aggressiveness, lymph node metastasis, and response to ^131^I therapy, enabling clinicians to tailor the most precise and individualized treatment for each patient on the basis of the individual microbiome (**Table 1**).

### 5.1 Predicting malignant tumors

Zhang et al. included 74 subjects, including 20 cases of TC, 18 cases of thyroid nodules, and 36 healthy controls. The composition of GM was analyzed by 16S rRNA gene sequencing. The results showed that the relative abundance of *Neisseria* (*P* < 0.001) and *Streptococcus* (*P* < 0.001) was significantly higher in TC patients than in healthy controls. *Butyricimonas* (*P* < 0.001) and *Lactobacillus* (*P* < 0.001) had significantly lower relative abundance. The study also found associations between the GM and indicators of disease. In TC patients, the levels of TSH and free triiodothyronine (FT3) were generally high, there was a significant positive correlation between TSH and *Porphyromonas* (r = 0.57, *P* < 0.01), and there was a positive correlation between FT3 and *Streptococcus* (r = 0.47, *P* < 0.05). In addition, FT3 was negatively correlated with *Lactobacillus* (r = 0.47, *P* < 0.05), and its relative abundance was slightly lower than that of the healthy controls. In summary, *Porphyromonas*, *Streptococcus* and *Lactobacillus* may be used as key biomarkers to identify thyroid nodules and cancer (14).

### 5.2 Predicting lymph node metastases

Yu et al. collected fecal specimens from 90 patients with TC and 90 healthy controls. The microbiota was analyzed by 16S rRNA gene sequencing. A predictive model consisting of 10 genera was found to distinguish TC patients from healthy controls. The 10-genus prediction model constructed by LEfSe analysis and lasso regression had an area under the curve (AUC) of 0.809 and 0.746 for TC patients and healthy controls in the exploration and validation cohorts, respectively. This feature includes elevated levels of *g Bacteroides*, *g Lachnoclostridium* and *g norank_f Lachnospiraceae*, while *g Prevotella_9*, *g Collinsella*, *g Faecalibacterium*, *g Dorea*, *g Ruminococcaceae_UCG-014*, *g Ruminococcaceae_UCG-002* and *g Su bdoligranulum* levels were reduced. Lymph node metastases are common in TC patients. In addition, Yu et al. found that microbial signatures of 4 genera may be able to distinguish TC patients with and without lymph node metastasis. A 4-genus model, including *g Hungatella*, *g Alistipes*, *g Fusobacterium*, and *g Phascolarctobacterium*, was generated using forward stepwise logistic regression with an AUC of 0.778 (18).

Dai et al. performed 16S rRNA gene sequencing on tumor tissues and matched paracancerous tissues of 30 TC patients and found that the richness and diversity of thyroid flora in TC tissues were lower than those in paracancerous tissues. The abundance of *Sphingomonas* and *Comamonas* was significantly increased in tumor tissues and adjacent tissues, respectively. The combined detection of *Sphingomonas* and *Comamonas* could distinguish tumor samples from peritumoral samples with an AUC of 0.981 (95%CI 0.949-1.000). In addition, *Sphingomonas* abundance could be used to identify lymph node metastasis, and the abundance of *Sphingomonas* in N1 stage was significantly higher than that in N0 stage. The AUC of *Sphingomonas* in distinguishing N0 from N1 stage was 0.964 (95%CI 0.907-1.000). From a clinical perspective, the thyroid microbiota differed between gender and histological subtypes, with *Micrococcus luteus* and *Bradyrhizobium Jordan* showing a significant positive correlation with MACIS score (distant metastasis, patient age, integrity of surgical resection, local invasion, and tumor size) (15).

### 5.3 Predicting invasiveness

Yuan et al. analyzed the ITM and its role in tumor progression using 16S rRNA sequences and found that a substantial microbial abundance was detected in tumor tissue from all PTC patients. Female patients have higher α diversity than male patients, which may be related to their higher susceptibility to TC. The α diversity index (Shannon and Simpson index, *P* = 0.0309 and *P* = 0.0088) of ITM in patients with T3/T4 (advanced stage) was significantly higher than that in patients with T1/T2 (relatively early stage). The study also identified 8 tumor bacterial taxa (*Pseudomonas*, *Rhodococcus*, *Sphingomonas*, *Streptococcus*, *Granulicatella*, *Haemophilus*, *g_unclassified_o_Rhizobiales*, and *g_norank_f_norank_o_Coriobacteriales*) with high predictive value for PTC invasion status (27).

### 5.4 Predicting tumor subtype

Gnanasekar A et al. performed microbiological analysis of PTC specimens from 563 TC patients. The ITM was also found to be different according to gender and tumor subtype. Microbial abundance in male samples was positively correlated with more chromosomal alterations and negatively correlated with tumor suppressor pathways, thus the prognosis of male PTC was worse. Hypercellular PTC is more aggressive than classical and follicular PTC, which may be related to the enrichment of *Micrococcus luteus* and *Bradyrhizobium* sp. *BTAi1*. In addition, abundant microbiota was highly correlated with immune-related genes such as CD4, CCL18, and CXCL14, and microorganisms significantly abundant in the hypercellular and male patient cohorts were also associated with higher mutant expression and methylation of tumor suppressor genes. *Frankia* sp. and *Anabaena* sp. *K119*, which were enriched in normal tissue samples of all PTC subtypes, were negatively correlated with pathological M stage (30).

### 5.5 Predicting response to ^131^I therapy

GM can be used as a predictor of treatment response in PTC patients before ^131^I treatment. Zheng et al. sequenced fecal samples before and after ^131^I treatment and found that butyric acid-producing *g_Dorea* was an independent predictor of response to ^131^I treatment. By increasing the relative abundance of *g_Dorea* and *g_Bifidobacterium* in the GM, It is expected to improve the response of postoperative PTC patients to ^131^I treatment (25).

### 5.6 Current limitations and future directions

Current studies are largely cross-sectional, confounded by diet, medications, and comorbidities, and lack methodological standardization across sample types and sequencing platforms. Longitudinal and multicenter validation data remain scarce, limiting causality inference and clinical generalizability. Future work should prioritize: 1) multi-omics integration with Mendelian randomization to establish causality; 2) engineered bacteria for tumor-targeted therapy; and 3) prospective interventional trials evaluating probiotics/FMT as adjuncts to RAI therapy, with defined endpoints and patient stratification by baseline microbiota.

## 6 Conclusions

This review highlights the roles of the gut and intratumoral microbiota in thyroid cancer (TC), supporting the “gut–thyroid axis”. In TC pathogenesis, key microbial dysbiosis drives tumor-promoting pathways: disrupted lipid metabolism elevates 27-hydroxycholesterol to activate estrogen receptors, gut microbial β-glucuronidase increases circulating estrogen to fuel papillary TC, and microbial flavonoid metabolites inhibit thyroid peroxidase to raise TSH levels. The critical targets involved include TPO, estrogen receptors, ANGPTL4, and bile acid signaling. For diagnosis, TC-associated microbial shifts (e.g., enrichment of *Bacteroidales* and *Clostridiales* during anti-PD-1 therapy) offer potential predictive biomarkers. For therapy, specific bacteria (e.g., *Akkermansia*, *Lactobacillus*) enhance anti-tumor immunity while others (e.g., *Bacteroidetes*) mitigate immune-related colitis, and genetically engineered bacterial vectors provide novel delivery platforms. Future work integrating multi-omics, engineered bacterial vectors, and rigorous interventional trials will accelerate the clinical translation of microbiota research in TC.
